# Altering the Immunogenicity of Hemagglutinin Immunogens by Hyperglycosylation and Disulfide Stabilization

**DOI:** 10.3389/fimmu.2021.737973

**Published:** 2021-10-07

**Authors:** Dana N. Thornlow, Andrew N. Macintyre, Thomas H. Oguin, Amelia B. Karlsson, Erica L. Stover, Heather E. Lynch, Gregory D. Sempowski, Aaron G. Schmidt

**Affiliations:** ^1^ Ragon Institute of MGH, MIT and Harvard, Cambridge, MA, United States; ^2^ Department of Microbiology, Harvard Medical School, Boston, MA, United States; ^3^ Duke Human Vaccine Institute, Duke University School of Medicine, Durham, NC, United States; ^4^ Departments of Medicine and Pathology, Duke University School of Medicine, Durham, NC, United States

**Keywords:** influenza, hemagglutinin, glycans, immunogen design, adaptive immunity, B cells

## Abstract

Influenza virus alters glycosylation patterns on its surface exposed glycoproteins to evade host adaptive immune responses. The viral hemagglutinin (HA), in particular the H3 subtype, has increased its overall surface glycosylation since its introduction in 1968. We previously showed that modulating predicted N-linked glycosylation sites on H3 A/Hong Kong/1/1968 HA identified a conserved epitope at the HA interface. This epitope is occluded on the native HA trimer but is likely exposed during HA “breathing” on the virion surface. Antibodies directed to this site are protective *via* an ADCC-mediated mechanism. This glycan engineering strategy made an otherwise subdominant epitope dominant in the murine model. Here, we asked whether cysteine stabilization of the hyperglycosylated HA trimer could reverse this immunodominance by preventing access to the interface epitope and focus responses to the HA receptor binding site (RBS). While analysis of serum responses from immunized mice did not show a redirection to the RBS, cysteine stabilization did result in an overall reduction in immunogenicity of the interface epitope. Thus, glycan engineering and cysteine stabilization are two strategies that can be used together to alter immunodominance patterns to HA. These results add to rational immunogen design approaches used to manipulate immune responses for the development of next-generation influenza vaccines.

## Introduction

Viruses use antigenic evolution to introduce glycans on their surface glycoproteins to evade immune surveillance by the host adaptive response; HIV and its envelope (Env) glycoprotein is one notable example (1). Indeed, for HIV Env, glycans account for approximately half the mass of the protein, and glycan density and heterogeneity can affect induction of broadly neutralizing antibodies (bnAbs) (1–3). Influenza (4, 5), ebola (6, 7), hepatitis C (8), Lassa (9–11), and coronavirus (12) are other examples of viruses that vary their glycosylation to “shield” conserved epitopes. Influenza has varied its hemagglutinin (HA) glycosylation profiles across subtypes since its introduction into the human population. Circulating H1 HAs have maintained approximately 8 glycans since their re-introduction in 1977 until the introduction of the pandemic H1N1 in 2009 which lost 4 glycans relative to previous historical H1s. H3 HAs, however, have increased overall glycans since the 1968 pandemic from 7 to 13.

Conserved epitopes on influenza HA are targets for broadly protective humoral immunity. These epitopes include the receptor binding site (RBS), stem, and “interface” regions (13). Antibodies that engage the RBS neutralize by impeding viral entry through sterically blocking attachment to its receptor, sialic acid. However, cross-reactive RBS-directed antibodies are subdominant, and their breadth is often dependent on having conserved features in their complementary determining region 3 (HCDR3) to present sialic acid-like contacts (14–20). The stem region of HA is similarly conserved, likely due to functional constraints necessary for undergoing conformational changes required for membrane fusion (21). Antibodies targeting the stem show breadth across nearly all HA subtypes (22–25). However, these antibodies targeting the stem are encoded by a relatively restricted set of variable genes and are difficult to elicit (21). More recently, we and others identified an additional conserved epitope at the HA trimer interface (26–30). This epitope lies at an otherwise occluded site between HA protomers in the native trimeric structure present on the virion surface. Nonetheless, antibodies targeting this epitope have been isolated from both human and murine sources and confer protection *via* an Fc-dependent mechanism (26–28). This implies that this occluded epitope is transiently exposed enough on the virion surface or the infected cell to be targeted by such antibodies (31).

Current immunogen design efforts for next generation influenza vaccines are focused on eliciting protective antibodies targeting these conserved epitopes (32). Here, we analyzed whether glycan engineering along with cysteine stabilizing mutations could modulate elicited humoral immune responses. We used the H3 A/Hong Kong/1/1968 HA as a model antigen to develop glycan shielded HA immunogens that block variable surface-exposed epitopes; we selectively exposed or concealed the RBS epitope with glycans and further stabilized the trimeric HA by introducing cysteine residues at both the head and stem interfaces. We immunized mice with stabilized or non-stabilized, glycan-modified immunogens and characterized elicited immune responses. We observed a reduction in serum responses to the stabilized immunogens, indicating that blocking surface epitopes with glycans or disulfide stabilization can significantly alter their overall immunogenicity. These data show how different protein engineering strategies can be combined to influence humoral responses to conserved epitopes on the influenza HA.

## Results

**Figure 1:**
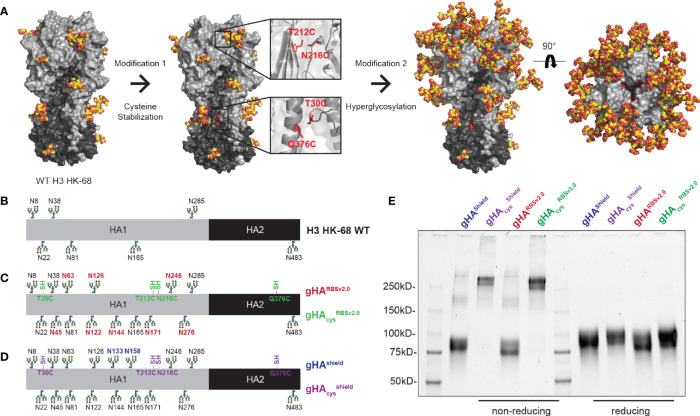
Design of second-generation hyperglycosylated hemagglutinin (gHA) immunogens with interface disulfide stabilization mutations. **(A)** Structural representation of H3A/Hong Kong/1/1968 (H3 HK-68) HA (PDB: 4FNK) with trimer-stabilizing interface cysteine mutations at residues 30, 212, 216, and 376, and additional surface glycans. **(B)** Linear representation of wild-type (WT) H3 HK-68 with native glycosylation sites marked. **(C)** Linear representation of gHA^RBSv2.0^ and gHA_cys_^RBSv2.0^ with additional glycans relative to WT marked in red, and cysteine stabilizing mutations marked in green. **(D)** Linear representation of gHA^Shield^ and gHA_cys_^shield^ with additional glycans relative to WT marked in blue, and cysteine stabilizing mutations marked in purple. **(E)** SDS-PAGE biochemical analysis of gHA immunogens under non-reducing and reducing conditions. Cysteine stabilized mutants show predominantly trimeric bands under non-reducing conditions (left) that collapse back to monomeric bands under reducing conditions (right).

Based on our previous work, we designed second-generation glycan-modified hemagglutinin (gHA) immunogens based on the historical H3A/Hong Kong/1/1968 HA (HK-68). Our first-generation gHAs included three immunogens with varying degrees of glycosylation: (1) an “RBS-exposed” immunogen (gHA^RBS^), in which the entire surface is hyperglycosylated but leaves the RBS exposed, (2) an “RBS-concealed” (gHA^cRBS^) immunogen with a single glycan at position 133 to abrogate binding of RBS-directed antibodies, and (3) a “full-shield” immunogen (gHA^shield^) which obscures all surface-exposed epitopes. For this study, we modified our initial gHA^RBS^ immunogen to include two additional predicted N-linked glycans (PNGs) at residues 122 and 126 to create gHA^RBSv2.0^; this construct differs from gHA^shield^ by two PNGs at residues 133 and 158, both in the RBS pocket (**Figure 1**). We used these two immunogens, gHA^RBSv2.0^ and gHA^shield^, to discern RBS- from non-RBS directed responses and as templates for further modifications.

Antibodies elicited by our previous immunization with first-generation gHAs focused predominantly to the trimer interface; therefore, we hypothesized that occluding the interface epitope, could re-focus the immune response to the RBS. This may have been a consequence of the engineered glycans destabilizing the HA trimer resulting in mixed trimer and monomeric HAs; the latter readily exposing the interface epitope (**Supplemental Figure 1**). To that end, we stabilized the HA trimer by engineering cysteines at residues 212 and 216 (33) (H3 numbering) on the HA head and at residues 30 and 376 (34) on the HA stem, in order to occlude immune responses directed to the HA interface (**Figure 1A**). These stabilizing mutations were engineered onto both gHA^RBSv2.0^ and gHA^shield^, to yield their cysteine-stabilized counterparts, referred to as gHA_cys_^RBSv2.0^ and gHA_cys_^shield^ (**Figures 1B**–**D**). We confirmed disulfide presence using SDS-PAGE under reducing and non-reducing conditions (**Figure 1E**). The cysteine modified hyperglycosylated HA proteins showed predominantly trimeric bands that collapsed into a monomeric species upon reduction. They also are stable trimers under native conditions, as confirmed by size-exclusion chromatography (**Supplemental Figure 1**).

**Figure 2:**
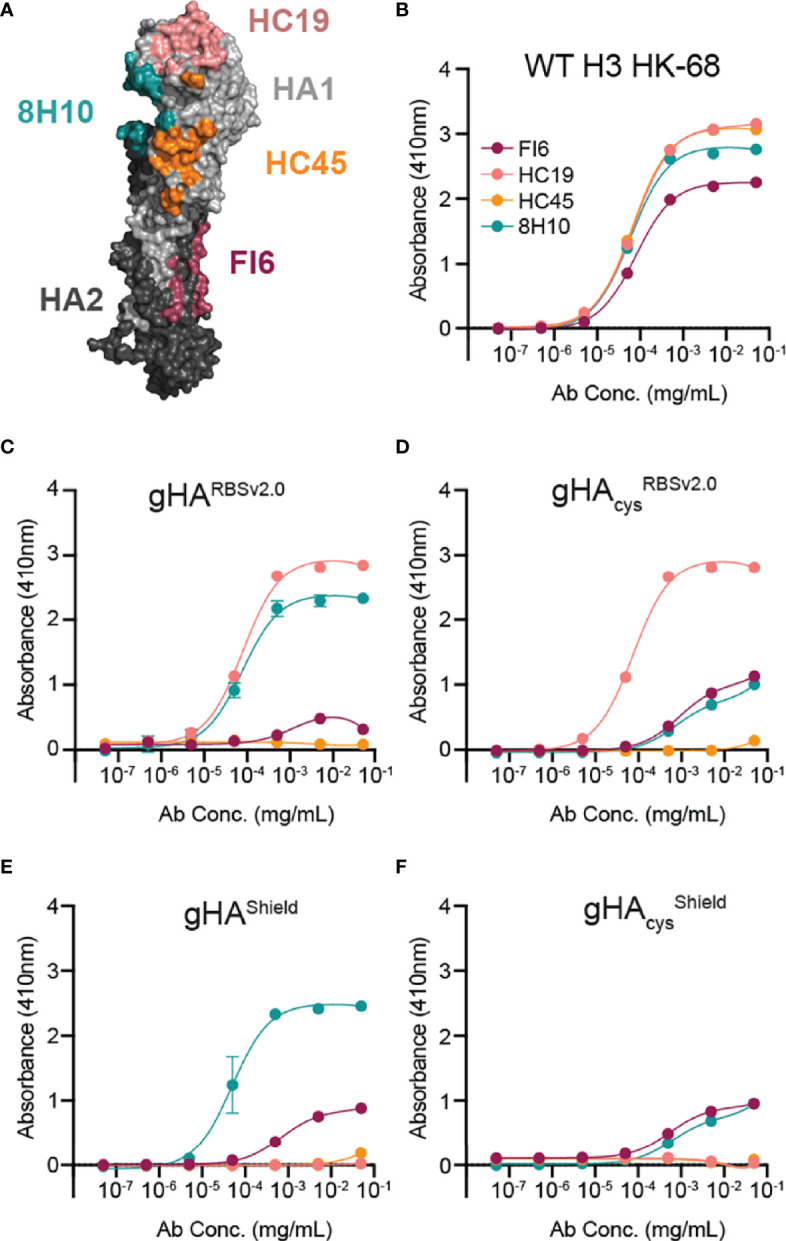
Characterization of gHA immunogens with structurally characterized antibody panel. **(A)** Antibody panel epitopes mapped onto WT H3 HK-68 monomer (PDB: 4WE4). Antibodies are representative of four major antigenic sites on HA: HC19 (pink; PDB: 2VIR; receptor binding site (RBS)), HC45 (orange; PDB: 1QFU; vestigial esterase domain), FI6 (maroon; PDB: 3ZTJ; stem), and 8H10 (teal; PDB: 6N5B; interface). **(B–F)** Selective placement of glycans and interface cysteines on gHA immunogens abrogate binding of epitope-specific antibodies as shown by ELISA. Binding curves of the antibody panel are shown against WT H3 HK-68 **(B)**, gHA^RBSv2.0^ **(C)**, gHA_cys_^RBSv2.0^ **(D)**, gHA^Shield^ **(E)**, gHA_cys_^shield^ **(F)**. Average absorbance at 410nm ± SD is reported for technical duplicates.

We next characterized all four gHA immunogens by ELISA using an assembled panel of structurally characterized, conformation-specific antibodies binding unique antigenic sites on HA, including HC19 (35), HC45 (36), FI6 (22), and 8H10 (27), which bind the RBS, vestigial esterase, stem, and interface epitopes, respectively (**Figure 2A**). All four gHA immunogens abrogated binding of HC45 and FI6. Both gHA^RBSv2.0^ immunogens engaged HC19 (**Figures 2C, D**), whereas gHA^shield^ immunogens abrogated binding by HC19 (**Figures 2E, F**), indicating that these immunogens selectively conceal or expose the RBS epitope, respectively. Additionally, both cysteine-stabilized immunogens, gHA_cys_^RBSv2.0^ and gHA_cys_^shield^, selectively abrogated binding of the interface-directed antibody, 8H10, characterized in our previous study (27); this confirms that the stabilized trimers effectively mask the interface epitope (**Figures 2D, F**). All immunogens engage the conformation-specific MEDI8852 antibody (37), that binds the stem in a different orientation relative to FI6, and confirms structural integrity of the stem epitope (**Supplemental Figure 2**).

**Figure 3:**
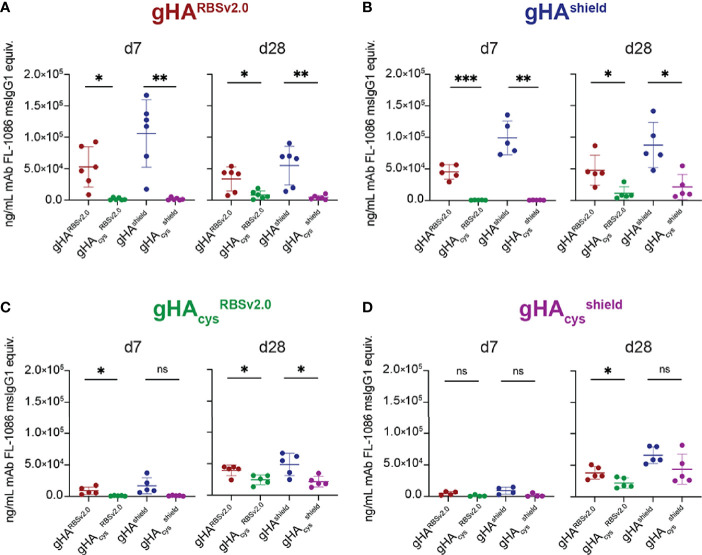
gHA immunogens elicit cross-immunogen reactivity. Plot titles indicate immunogen and x-axis indicates Luminex antigen. At day 7 (d7), gHA^RBSv2.0^ (n=6) **(A)** and gHA^Shield^ (n=5) **(B)** immunogens elicit interface-directed serum responses, while the cysteine-stabilized counterpart immunogens **(C, D)** elicit a nearly undetectable serum response. By day 28 (d28, right), cysteine-stabilized gHA_cys_^RBSv2.0^ (n=5) **(C)** and gHA_cys_^shield^ (n=5) **(D)** immunogens elicit a weak, but present serum response to all immunogens. All serum responses are normalized to a control antibody, FL-1086, that binds all four immunogens, and reported as ng/mL FL-1086 msIgG1 equivalents ± SD; n = number of mice used. Statistical significance was determined by a student’s t-test with Welch’s correction. (*P < 0.05; **P < 0.01; ***P < 0.001; ns, not significant.

We then assessed the immunogenicity and antigenicity of gHA^RBSv2.0^, gHA_cys_^RBSv2.0^, gHA^shield^, and gHA_cys_^shield^. We immunized mice and collected serum at day 0, 7, 14, 21, and 28. Serum IgG responses were assessed *via* multiplex bead array (Luminex platform) for binding to each of the four immunogens (**Figure 3** and **Supplemental Figure 3**). While the gHA^RBSv2.0^ and gHA^shield^ immunogens elicited a robust self-directed serum response, there was a comparatively weak reactivity to the cysteine modified counterparts indicating a predominantly interface-directed response (**Figures 3A, B**). This pattern persisted throughout the course of the experiment (**Supplemental Figure 3**). These data are consistent with our previous observations of the role of glycan shielding and immunodominance of the interface epitope (27). While immunizations with cysteine gHA_cys_^RBSv2.0^ and gHA_cys_^shield^ elicited a nearly undetectable response at day 7, by day 28 we observe a similar overall magnitude of response comparable to the non-cysteine stabilized immunogens (**Figures 3C, D** and **Supplemental Figure 3**). However, unlike gHA^RBSv2.0^ and gHA^Shield^, this response was comparable across all four immunogens for both gHA_cys_^RBSv2.0^ and gHA_cys_^shield^. This potentially indicates that serum responses elicited by disulfide-stabilized gHAs were directed towards a common epitope exposed across all four immunogens. Notably, we did not observe differential reactivity to our “RBS-exposed” and “shield” immunogens at any timepoint, indicating that there was likely not a robust RBS-directed humoral response elicited by our exposed RBS immunogens. Overall, these data indicate that the combination of hyperglycosylation and disulfide stabilization alters the immunogenicity kinetics of gHA immunogens but cannot significantly redirect humoral responses to the exposed RBS epitope.

**Figure 4:**
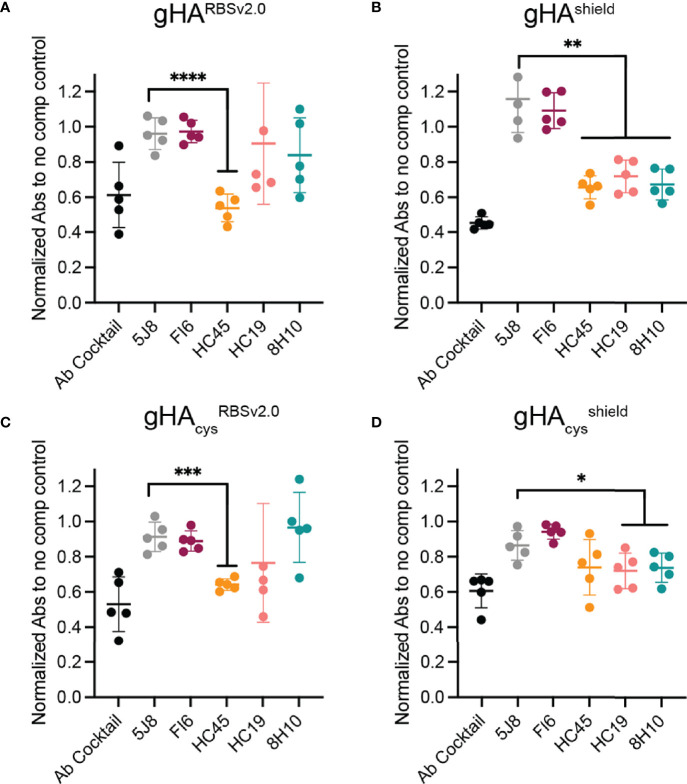
Epitope mapping gHA elicited serum response. A serum competition ELISA with structurally characterized antibodies was used to epitope map elicited serum responses by gHA immunogens: gHA^RBSv2.0^ **(A)**, gHA^shield^ **(B)**, gHA_cys_^RBSv2.0^ **(C)** and gHA_cys_^shield^ **(D)**. Day 28 serum from gHA^RBSv2.0^ **(A)**, gHA^shield^ **(B)**, and gHA_cys_^RBSv2.0^ **(C)** elicited a significant HC45-like response. None of the gHA immunogens elicited a significant FI6-like response. Statistical significance was determined by a student’s t-test with Welch’s correction relative to negative control (5J8). (*P < 0.05; **P < 0.01; ***P < 0.001; ****P < 0.0001).

Next, we performed competition ELISAs with a panel of conformational specific antibodies with d28 serum to epitope map the elicited responses to our immunogens (**Figure 4**). Our antibody panel included the FI6 (stem), HC45 (vestigial esterase), HC19 (RBS), and 8H10 (interface) epitopes (**Figure 2**). The H1 RBS-directed 5J8 (38), which does not bind to H3 HK-68, was included as a negative control. Serum from gHA^RBSv2.0^, gHA_cys_^RBSv2.0^, and gHA_cys_^shield^ showed significant competition with HC45 (**Figures 4A**–**C**), that was not present in gHA_cys_^shield^ (**Figure 4D**). While not as significant as the HC45-like response, both gHA_cys_^RBSv2.0^, and gHA_cys_^shield^ immunogens apparently elicited a HC19-like and 8H10-like response, despite the presence of glycans and inter-protomer disulfides. There was no FI6-like response detected in sera from any of the immunogens; this potentially indicates that despite fewer glycans present to obscure responses targeting the stem, this region remains subdominant.

**Figure 5:**
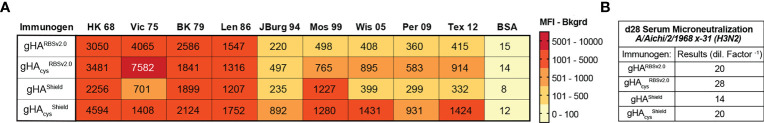
gHA immunogens elicit serum responses with limited breadth and neutralization. **(A)** Day 28 serum binding against a Luminex panel of antigenically distinct, historical H3 HAs spanning 1975-2012. Serum responses show limited breadth beyond H3Leningrad/360/1986 (x-91) (Len 86). Serum binding is reported as the average mean fluorescence intensity (MFI) minus background for all mice in a specific cohort. **(B)** Microneutralization of day 28 serum against matched A/Aichi/2/1968 X-31 (H3N2) virus. Endpoint titers are reported as the greatest dilution observed to exhibit ≥50% virus neutralization and are reported as geometric mean of duplicate dilution series. Day 28 serum from each immunogen show weak microneutralization of matched virus.

Finally, we analyzed day 28 serum for breadth and influenza virus neutralization activity. We assessed H3 breadth using a panel of antigenically distinct, historical H3s spanning 1968-2012 (**Figure 5** and **Supplemental Figure 4**. Serum IgG from all four immunogens bound to H3 from A/Hong Kong/1/1968, A/Victoria/3/1975, A/Bangkok/1/1979, and A/Leningrad/360/1986, with little to no binding beyond that point (**Figure 5A**). Additionally, serum elicited by all four immunogens had weak neutralization activity, with no appreciable difference between “RBS-exposed” and “shield” immunogens (**Figure 5B**). This is consistent with the above data that these immunogens do not elicit robust RBS-directed serum responses that would contribute to neutralization. Furthermore, the lack of neutralization by serum from gHA^RBSv2.0^ and gHA^shield^ immunogens is likely a consequence of a focused response to HC45-like or interface epitopes that do not contribute to neutralization.

## Discussion

In this work, we determined whether modifying HA using hyperglycosylation and disulfide stabilization could focus antibody responses to the subdominant RBS epitope. We hypothesized that by both shielding surface-exposed epitopes and by restricting access to the immunodominant interface epitope by glycans, theoretically the only accessible epitope is the RBS, and thus should become immunodominant, or at minimum, be able to elicit a detectable RBS-directed response. This epitope masking approach has been effective in focusing responses to the CD4 binding site on HIV-1 Env (39). Additionally, inter-protomer disulfide stabilization has been an effective method for improving or focusing immune responses to RSV (40, 41), norovirus (42), parainfluenza virus (43), and HIV (44). To our knowledge, however, this is the first example to study the effect of inter-protomer disulfide stabilization of full-length influenza A HA in combination with hyperglycosylation on immunogenicity. However, in this study we showed that obscuring all surface-exposed epitopes except the RBS did not robustly direct responses to the RBS.

The observed lack of an RBS-directed serum response could be due to a variety of factors. Human-derived bnAbs that engage the RBS of group 1 and group 2 HAs have been isolated and appear to be relatively abundant (14–17, 19, 20). These RBS-directed antibodies require long HCDR3s (~19 amino acid in length) to engage the recessed RBS. Because the C57BL/6 mice used in this study tend to have shorter HCDR3s, it is likely that this restricts humoral responses targeting the RBS epitope. Additionally, these antibodies could be rare in the mouse antibody repertoire, and thus may not be readily detected in serum. Alternative murine models with human variable heavy genes knocked-in [*e.g.*, JH6 (45)] may be better suited for characterizing immunogens designed to elicit RBS-directed responses.

Despite being nearly “immune silent” at early time points, the disulfide-stabilized immunogens eventually elicited a weak, but detectable, antibody response at days 21 and 28. This may indicate that the engineered glycans do not completely shield surface-exposed epitopes. In other words, it is possible that antibodies target “holes” in the engineered glycan shield that may ultimately contribute to the humoral response. From competition ELISA data, this could be an epitope that partially overlaps with HC45 within the vestigial interface region but is not obscured by glycans on gHA constructs, or another epitope(s) not covered by our antibody panel. A similar phenomenon has been observed in HIV-1, where antibodies targeting the CD4 binding site on gp120 such as VRC01-like antibody, VRC08 (46), as well as N6 (47), could evolve around a glycan at position N276, present on ~95% of circulating HIV-1 strains. Future immunogen design iterations may vary the pattern of engineered glycans to optimize placement and shielding.

Overall, these data show how hyperglycosylation and disulfide stabilization can effectively modulate humoral responses. There appears to be a “limit” on available surface-exposed epitopes on our immunogens that can be effectively shielded by glycans to dampen overall humoral response. This parallels how viruses use glycosylation of their envelope proteins to evade host adaptive immune responses [e.g., HIV (1–3), ebola (6), hepatitis C (8), influenza (4, 5)]. It remains to be seen whether hyperglycosylation alone or in combination with disulfide stabilization can be leveraged to elevate a subdominant epitope to dominance, in particular the RBS. While in wildtype mice this was not achieved, the immunogens designed and characterized here may indeed immune focus to the RBS in humans who can more readily elicit RBS-directed responses.

## Materials and Methods

### Hyperglycosylated HA Immunogen Expression and Purification

Hyperglycosylated hemagglutinin (gHA) protein immunogens were cloned into pVRC vectors for mammalian cell (HEK/Expi 293F) production in HEK293F or Expi 293F suspension cells. DNA constructs were confirmed by sequencing at Genewiz. HA constructs were expressed with a C-terminal 3C HRV-cleavable T4-fibritin (foldon) tag (for trimerization) and Streptavidin binding protein (SBP)-His_8X_ affinity tag (for purification). Proteins were purified from supernatants on a Cobalt-TALON resin, followed by gel filtration chromatography on a Superdex 200 column (GE Healthcare) in Dulbecco’s Phosphate-Buffered Saline (D-PBS) (pH 7.5). Foldon and affinity tags were removed with His-tagged HRV 3C protease at 4°C overnight, then passed over a Cobalt-TALON resin to remove protease, cleaved tags, and non-cleaved protein. Purified, cleaved proteins were concentrated using Amicon^®^ ultra centrifugal filters, then quantified using 280 nm absorbance.

### Recombinant HA Expression and Purification

Recombinant full-length, soluble ectodomains of H3 A/Moscow/10/1999, A/Victoria/3/1975, A/Johannesburg/33/1994, A/Bangkok/1/1979, A/Perth/16/2009 and A/Leningrad/360/1986. H3 HAs were cloned into pFastBac vectors. Expression constructs contained a C-terminal His_6x_ tag, for purification, and T4-fibritin foldon tag, for trimerization. Baculoviruses were collected from Sf9-transfected cells and used to infect Sf9-cultured cells grown in Sf-900II SFM medium (Life Technologies). rHAs were then purified *via* Ni-NTA column three days post-infection and used in Luminex assays.

Recombinant full-length, soluble ectodomains of H3 A/Hong Kong/1/1968, A/Wisconsin/67/2005 and A/Texas/50/2012 were cloned into pVRC vectors containing a C-terminal 3C HRV-cleavable T4-fibritin (foldon) tag (for trimerization) and Streptavidin binding protein (SBP)-His_8X_ affinity tag (for purification). Proteins were purified from supernatants 5 days post-transfection on a Cobalt-TALON resin, followed by gel filtration chromatography on a Superdex 200 column (GE Healthcare) in Dulbecco’s Phosphate-Buffered Saline (D-PBS) (pH 7.5). Purified rHAs were then used in Luminex and ELISA assays.

### Fab and IgG Expression and Purification

Variable domain genes for IgG production were codon optimized and synthesized by Integrated DNA Technologies (IDT) and subcloned into pVRC vectors containing humanized heavy and light constant domains for expression in mammalian cells (HEK/Expi 293F cells). DNA constructs were sequenced confirmed using Genewiz. Humanized HC19 (35), HC45 (36), FI6 (22) and 8H10 (27) IgGs were expressed transiently in suspension 293F cells using polyethyleneamine (PEI) or ExpiFectamine 293 for 5 days. After 5 days, IgGs were purified from clarified supernatants on a Protein G resin, and eluted into Tris-Glycine buffer with 150mM NaCl.

### Size Exclusion Chromatography

To assess the oligomeric state(s) of wild-type H3 X31 HA and gHAs, ~200µg of purified protein with foldon trimerization tag was passed over a Superdex 200 10/300 increase column (GE Healthcare) in Dulbecco’s Phosphate-Buffered Saline (D-PBS) (pH 7.5). The foldon tag was then removed using His-tagged HRV 3C protease overnight at 4°C overnight. The cleavage reaction was then re-purified over a cobalt-TALON resin to remove non-cleaved HA, cleaved foldon tag, and protease. The cleaved HA was passed over the same Superdex 200 10/300 increase column. All traces were normalized to their respective peak absorbance value and plotted using Prism 9 (Graphpad).

### ELISA

100 ng of hyperglycosylated or wild-type recombinant hemagglutinin proteins were adhered to high-capacity binding, clear bottom, 96-well plates (Corning) in PBS overnight at 4°C. Plates were blocked with 1% BSA in PBS containing 0.1% Tween-20 (PBS-T) for 1 hour at room temperature (RT), shaking. Blocking solution was removed, and plates were incubated with 10-fold dilutions of structurally characterized IgGs in PBS for 1 hour at RT, shaking. After 1 hour, plates were washed three times with PBS-T, then incubated with 1:20,000 diluted HRP-conjugated anti-human IgG (Abcam) for 1 hour at RT. Plates were washed three times with PBS-T. 1-Step ABTS substrate (ThermoFisher) was added to plates for 20-30 mins at RT, then stopped with 100 µL of 1% SDS. Absorbance measurements from each well at 410 nm were recorded using a plate reader. Data were plotted using Prism 9 (Graphpad) and relative binding was determined.

### Mouse Immunizations

C57BL/6 (female, 8-10wk) mice, obtained from Jackson Labs, were immunized with 10 µg of low to endotoxin-free gHA^RBSv2.0^ (n=6), gHA_cys_^RBSv2.0^ (n=5), gHA^shield^ (n=5), and gHA_cys_^shield^ (n=5) mixed 1:1 with Addavax (Invivogen) adjuvant, or adjuvant only control, *via* the IM route. Serum samples were prepared at day 0 (pre-bleed), 7, 14, 21, and 28 for Luminex bead-based multiplex binding analysis.

### Luminex Multiplex Assays

Hyperglycosylated HA (gHA), recombinant WT HA (rHA) or bovine serum albumen (BSA; Sigma – negative control) were conjugated to magnetic microspheres (Luminex Corp.) by carbodiimide coupling according to the manufacturer’s protocol. For relative quantitation of rHA-binding IgG, rHA- and BSA-coated microspheres (1500 of each) were mixed in 96-well plates with samples diluted in PBS, 1% BSA pH7.4 (assay buffer; Life Technologies), incubated for 1 hour at room temperature on an orbital shaker and then washed in assay buffer. Microspheres were then incubated with 4 μg/mL phycoerythrin (PE)-conjugated goat anti-mouse IgG (BioLegend) for 30 minutes, washed, and the PE median fluorescence intensity (MFI) of each microsphere population measured using a Bio-Plex 200 analyzer (BioRad). For relative quantitation of hyperglycosylated antigen-binding IgG, samples were incubated with antigen- and BSA-coated microspheres as above. Washed microspheres were then incubated with 4 μg/mL biotinylated goat anti-mouse IgG (BioLegend) in assay buffer for 30 minutes, washed, and incubated with 8 μg/mL Streptavidin-PE (Invitrogen) for 30 minutes. Microspheres were then washed and the PE MFI of each microsphere population measured using a Bio-Plex 200 analyzer. Serially diluted IgG1 isoform of a control monoclonal antibody (mAb) FL-1086 (25), which binds all gHA immunogens, were assayed alongside the samples and then Bio-Plex Manager v6.2 software (BioRad) was used to generate standard curves of MFI *versus* monoclonal concentration. These curves were used to convert sample MFIs to FL-1086-equivelants.

### Competition Serum ELISA

100 ng per well of wild-type (WT) recombinant hemagglutinin (rHA) proteins were adsorbed to high-capacity binding, clear bottom, 96-well plates (Corning) in PBS overnight at 4°C, as described above. Plates were blocked with 1% BSA in PBS containing 0.1% Tween-20 (PBS-T) for 1 hour at room temperature (RT), shaking. Blocking solution was removed, and 150ng/µL of humanized individual competing antibodies or antibody cocktail was added to each well and allowed to incubate for 1hr at RT, shaking. Next, a 1:250 dilution of day 28 serum in PBS was added on top of the competing antibody, for a final dilution of 1:500, then incubated for 1hr at RT, shaking. Plates were then washed three times with PBS-T, followed by incubation for 1hr at RT with HRP-conjugated, human/bovine/horse adsorbed anti-mouse secondary antibody (Southern Biotech) at a concentration of 1:4000. Plates were again washed three times with PBS-T, then developed, as above with ABTS substrate.

### Microneutralization

Microneutralization (MN) assays were used, as described (48–51), to determine serum endpoint titers against H3 A/Aichi/2/1968 (X-31) virus. All samples were assayed as duplicate dilution series. Endpoint titers are reported as the greatest dilution observed to exhibit ≥ 50% virus neutralization.

### Statistical Analyses

Statistical analyses described in the manuscript were performed using GraphPad Prism (v9.2.0). All comparisons were made using a parametric, unpaired student’s t-test with Welch’s correction with statistical significance denoted with the following asterisk key: * = P<0.05; ** = P<0.01; *** = P<0.001; **** = P<0.0001; ns = not significant.

## Data Availability Statement

The raw data supporting the conclusions of this article will be made available by the authors, without undue reservation.

## Ethics Statement

The animal study was reviewed and approved by Duke University Institutional Animal Care and Use Committee.

## Author Contributions

DT, AS, GS, and AK designed research. DT, AM, ES performed research. DT and AS analyzed data. DT and AS wrote the paper. DT, AS, GS, AM, and AK edited and commented on the paper. All authors contributed to the article and approved the submitted version.

## Funding

We acknowledge support from R01 AI146779 and P01 AI089618 (to AS). This research has been funded in whole or part with federal funds under a contract from the National Institute of Allergy and Infectious Diseases, NIH contract 75N93019C00050. Multiplex binding and microneutralization assays were performed in the Immunology and Virology Units, respectively, of the Duke Regional Biocontainment Laboratory, which received partial support for construction from the NIH/NIAID (UC6 AI058607; to GS).

## Conflict of Interest

The authors declare that the research was conducted in the absence of any commercial or financial relationships that could be construed as a potential conflict of interest.

The handling Editor declared a past co-authorship with one of the authors AS.

## Publisher’s Note

All claims expressed in this article are solely those of the authors and do not necessarily represent those of their affiliated organizations, or those of the publisher, the editors and the reviewers. Any product that may be evaluated in this article, or claim that may be made by its manufacturer, is not guaranteed or endorsed by the publisher.

## Supplementary Material

Supplementary Figure 1 Hyperglycosylation increases tendency to present “exposed” HA trimer interface epitope- Wild-type (WT) H3 HK-68 and gHAs were analyzed *via* size-exclusion FPLC for conformational changes pre- and post-Foldon (Fd) trimerization tag cleavage. WT **(A)** and cysteine-stabilized gHA **(B)** showed a small shift in elution volume (<0.5mL) due to the small change in molecular weight following Fd tag cleavage. gHA^RBS^ **(C)** and gHA^shield^ **(D)** showed an additional elution volume shift of ~1.5mL, indicating the presence of dimer or monomeric HA and an “exposed” interface epitope.

Supplementary Figure 2 MEDI8852 antibody engages gHA_cys_^shield^ immunogen, **(A)** Antibody panel epitopes mapped onto WT H3 HK-68 monomer (PDB: 4WE4). Antibodies are representative of epitopes on HA: HC19 [pink; PDB: 2VIR; receptor binding site (RBS)], HC45 (orange; PDB: 1QFU; vestigial esterase domain), FI6 (maroon; PDB: 3ZTJ; stem), MEDI8852 (sky blue; PDB: 5JW4; stem), and 8H10 (teal; PDB: 6N5B; interface). **(B)** WT H3 HK-68 engages all major epitope-directed antibodies in an ELISA format. **(C)** gHA^RBS^ engages interface (8H10), RBS (HC19) and stem directed MEDI8852. **(D)** ELISA show abrogated binding of all major epitope-directed antibodies to gHA_cys_^shield^, with the exception of MEDI8852.

Supplementary Figure 3 Time course of serum responses elicited by gHA immunogens - Plot titles indicate immunogen and x-axis indicates Luminex antigen.

Supplementary Figure 4 Day 28 serum elicited by gHA immunogens have limited breadth against a panel of antigenically distinct, historical H3 HAs. Day 0 (open circle) and day 28 (closed circle) serum from each individual mouse are plotted as background-subtracted MFI for each immunogen. Titles indicate Luminex antigen and X-axis titles indicate immunogens gHA^RBSv2.0^ (n=6), gHA_cys_^RBSv2.0^ (n=5), gHA^shield^ (n=5), and gHA_cys_^shield^ (n=5). Mean fluorescence intensity (MFI) signal minus background ± SD is reported; n = number of mice used.
