# Supplementary figures and images for: Altering the Immunogenicity of Hemagglutinin Immunogens by Hyperglycosylation and Disulfide Stabilization
Source: Front Immunol. 2021 Oct 7;12:737973. doi: 10.3389/fimmu.2021.737973 (PMC8528956; doi:10.3389/fimmu.2021.737973)

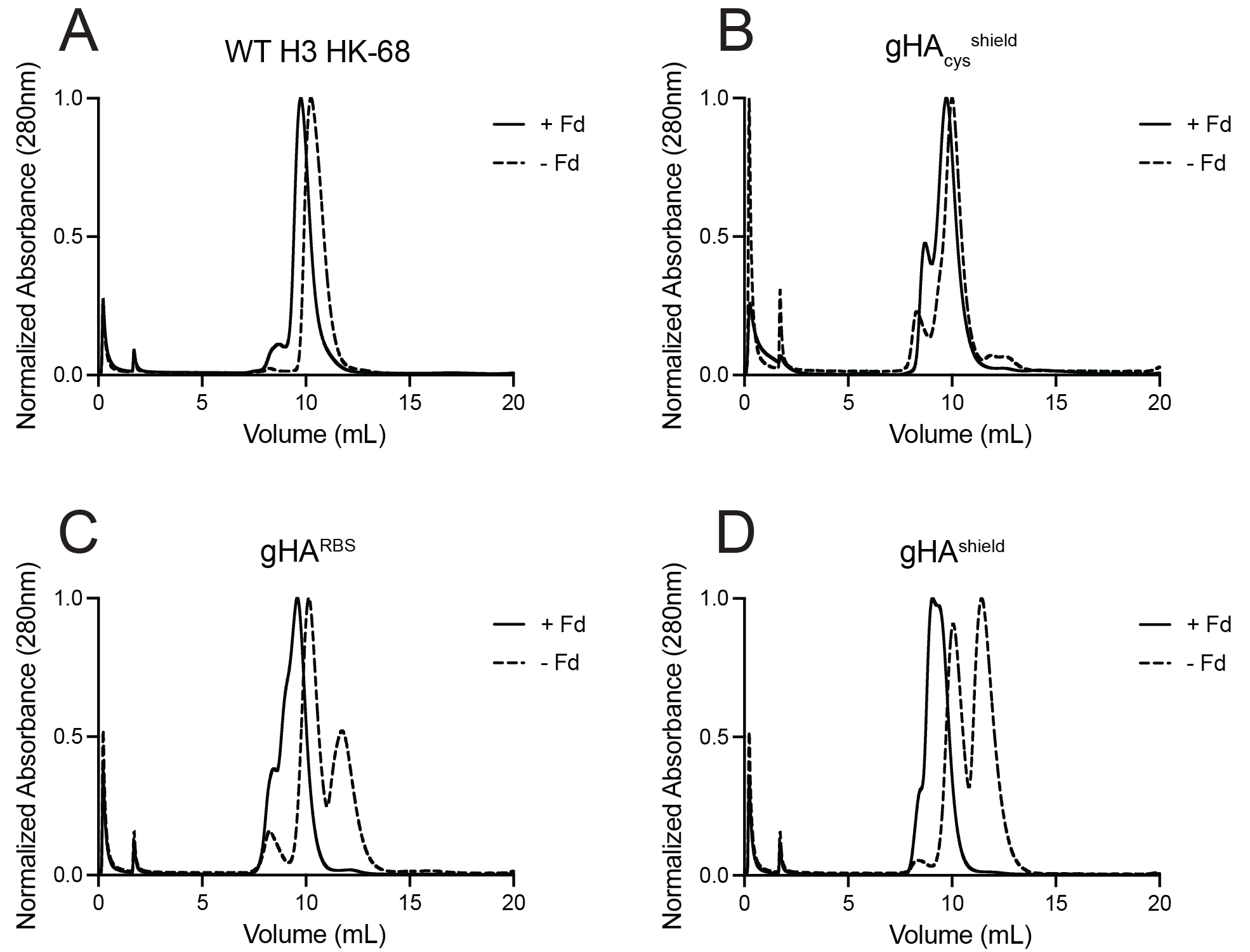

Supplement: Supplementary Figure 1 — Hyperglycosylation increases tendency to present “exposed” HA trimer interface epitope- Wild-type (WT) H3 HK-68 and gHAs were analyzed via size-exclusion FPLC for conformational changes pre- and post-Foldon (Fd) trimerization tag cleavage. WT (A) and cysteine-stabilized gHA (B) showed a small shift in elution volume (<0.5mL) due to the small change in molecular weight following Fd tag cleavage. gHARBS (C) and gHAshield (D) showed an additional elution volume shift of ~1.5mL, indicating the presence of dimer or monomeric HA and an “exposed” interface epitope. [file Image_1.tif]

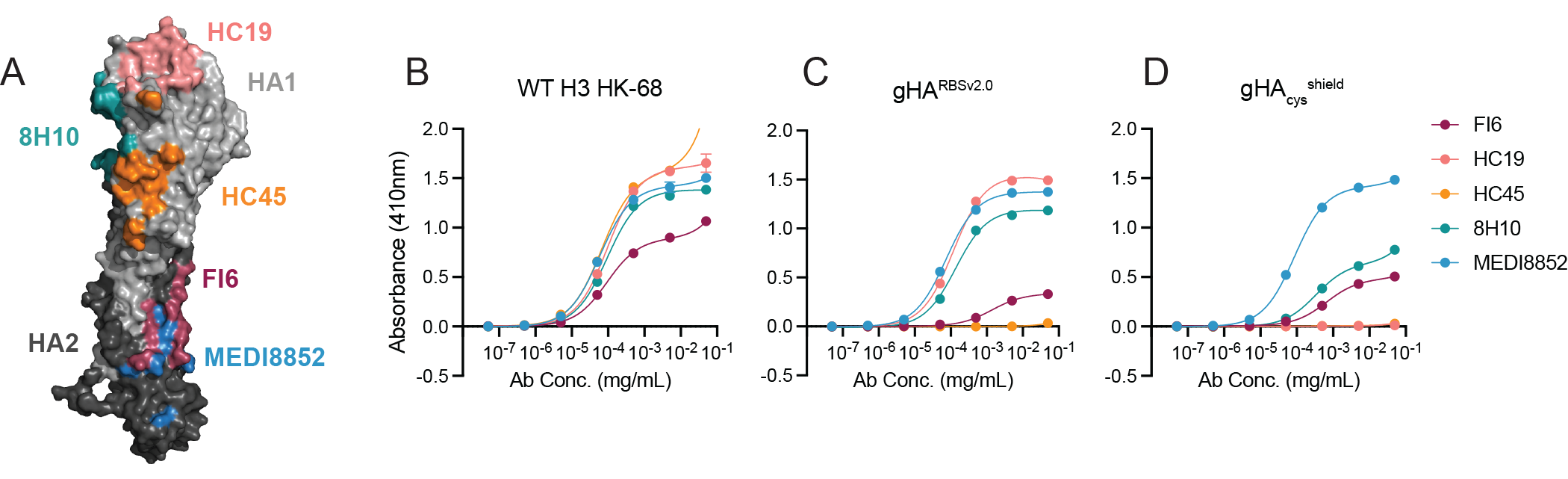

Supplement: Supplementary Figure 2 — MEDI8852 antibody engages gHAcys shield immunogen, (A) Antibody panel epitopes mapped onto WT H3 HK-68 monomer (PDB: 4WE4). Antibodies are representative of epitopes on HA: HC19 [pink; PDB: 2VIR; receptor binding site (RBS)], HC45 (orange; PDB: 1QFU; vestigial esterase domain), FI6 (maroon; PDB: 3ZTJ; stem), MEDI8852 (sky blue; PDB: 5JW4; stem), and 8H10 (teal; PDB: 6N5B; interface). (B) WT H3 HK-68 engages all major epitope-directed antibodies in an ELISA format. (C) gHARBS engages interface (8H10), RBS (HC19) and stem directed MEDI8852. (D) ELISA show abrogated binding of all major epitope-directed antibodies to gHAcys shield, with the exception of MEDI8852. [file Image_2.tif]

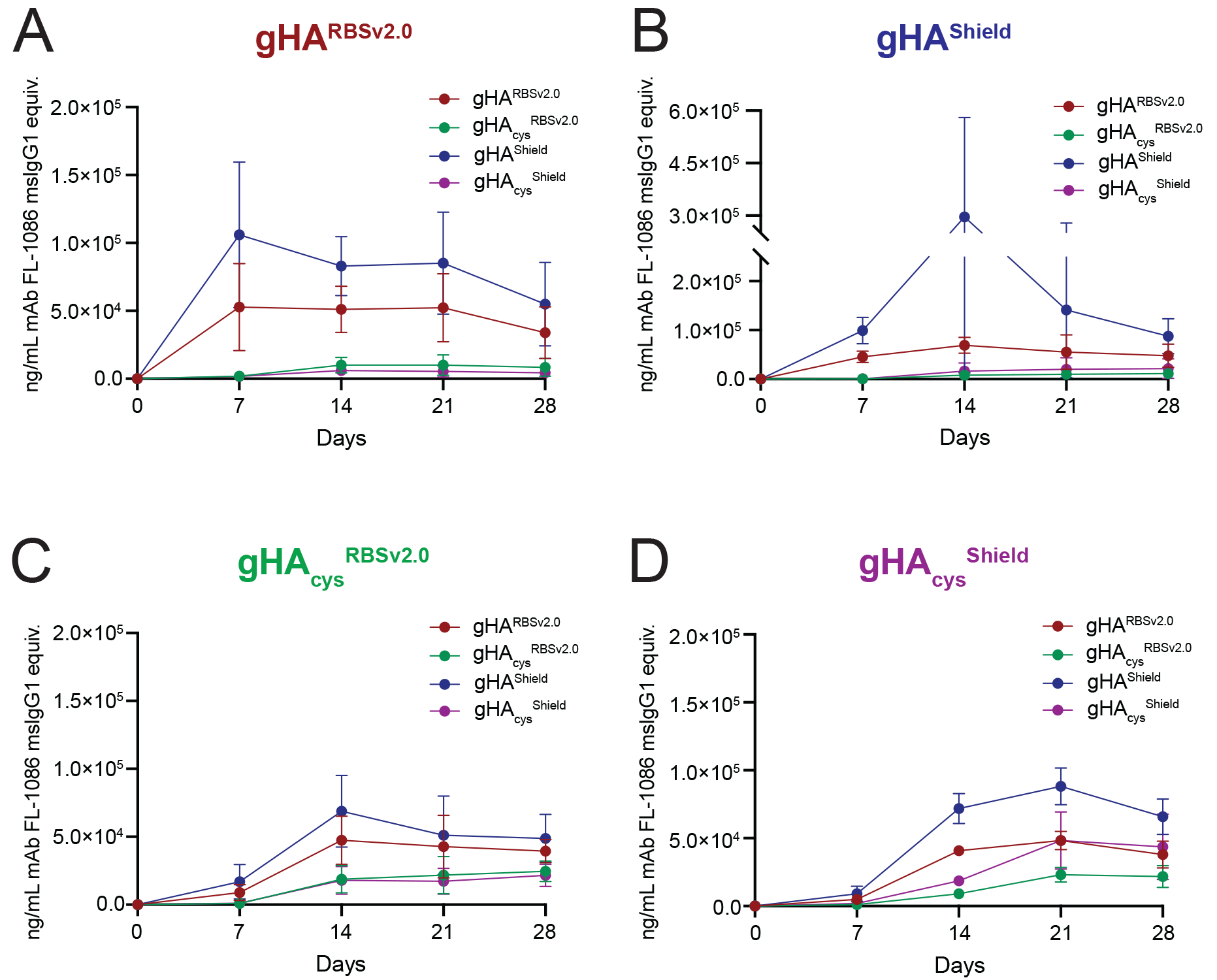

Supplement: Supplementary Figure 3 — Time course of serum responses elicited by gHA immunogens - Plot titles indicate immunogen and x-axis indicates Luminex antigen. [file Image_3.tif]

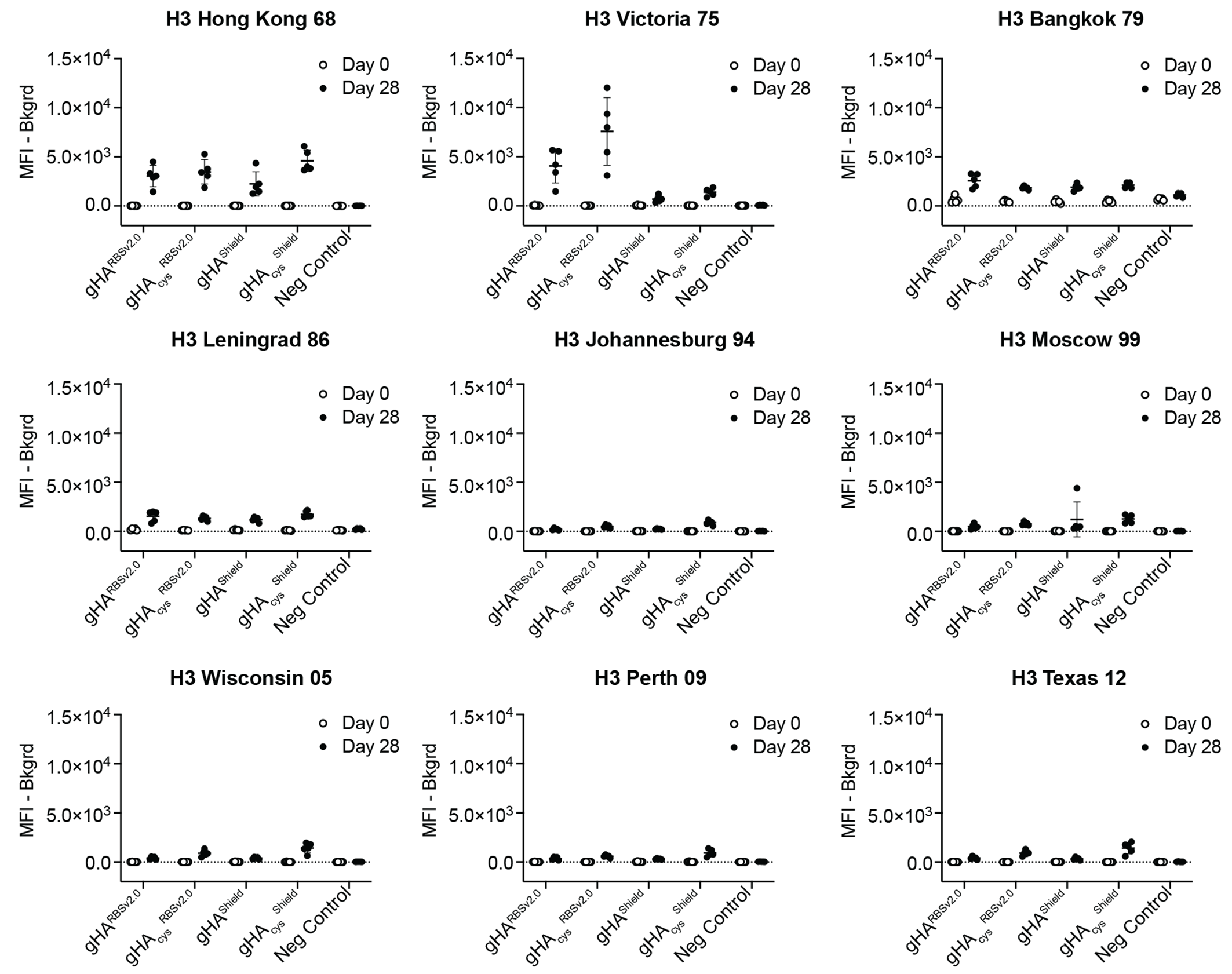

Supplement: Supplementary Figure 4 — Day 28 serum elicited by gHA immunogens have limited breadth against a panel of antigenically distinct, historical H3 HAs. Day 0 (open circle) and day 28 (closed circle) serum from each individual mouse are plotted as background-subtracted MFI for each immunogen. Titles indicate Luminex antigen and X-axis titles indicate immunogens gHARBSv2.0 (n=6), gHAcys RBSv2.0 (n=5), gHAshield (n=5), and gHAcys shield (n=5). Mean fluorescence intensity (MFI) signal minus background ± SD is reported; n = number of mice used. [file Image_4.tif]
